# Interactions Between Plant Proteins and Gut Microbiota as Determinants of Intestinal Health

**DOI:** 10.3390/microorganisms14030540

**Published:** 2026-02-26

**Authors:** Aleksandra Szydłowska, Barbara Sionek, Danuta Kołożyn-Krajewska

**Affiliations:** 1 Department of Food Gastronomy and Food Hygiene, Institute of Human Nutrition Sciences, Warsaw University of Life Sciences (WULS), Nowoursynowska St. 159C, 02-776 Warsaw, Poland; 2 Department of Dietetics and Food Studies, Faculty of Science and Technology, Jan Dlugosz University in Czestochowa, Al. Armii Krajowej 13/15, 42-200 Czestochowa, Poland

**Keywords:** plant proteins, gut microbiota, intestinal health

## Abstract

Plant proteins are an important component of the human diet and play a key role in shaping the composition and activity of the intestinal microbiota. Increasing evidence shows that interactions between plant-derived protein fractions and intestinal microorganisms have a significant impact on intestinal barrier function, immune response, and host metabolism. Undigested residues of proteins and peptides may constitute a substrate for intestinal bacteria, leading to the formation of metabolites with beneficial or harmful effects. On the one hand, fermentation products can support intestinal homeostasis through the synthesis of short-chain fatty acids or modulation of inflammatory responses; on the other hand, some compounds resulting from bacterial proteolysis may disturb the integrity of the intestinal epithelium. This article presents the current state of knowledge regarding the characteristics of plant-based proteins, their impact on the intestinal microbiota, and the importance of these interactions for intestinal health.

## 1. Introduction

In addition to carbohydrates and fat, proteins are one of the basic nutrients necessary to maintain life. They play a crucial role in performing various biological processes in a wide range of organisms. Among other things, they are essential for the proper growth and development of the body, replenishment of natural defects, tissue repair, and production of digestive and metabolic enzymes. Proteins also serve as substrates in the synthesis of many hormones and biologically active compounds, such as adrenaline and noradrenaline, which are thyroid hormones (thyroxine and triiodothyronine). Moreover, some proteins may also exhibit antimicrobial properties and additionally show antioxidant activity [1,2,3,4].

Protein is a fundamental macronutrient in the human diet. Chemically, proteins are composed of carbon, oxygen, nitrogen, hydrogen, sulfur, and phosphorus. We distinguish between simple proteins, composed primarily of amino acids, and complex proteins, which contain other components in addition to amino acids. The biochemical activity of proteins and their properties depend on their individual structure, shape, size, and interactions with other molecules [5].

The concept of protein requirement encompasses both total nitrogen and essential amino acid requirements. Therefore, the content and utilization of essential amino acids by the body can be considered valuable criteria for assessing dietary protein quality. Therefore, the assessment of protein’s biological value employs an amino acid-based approach, which involves comparing the essential amino acid content of dietary protein with a reference standard—a reference protein assumed to meet essential amino acid requirements at a protein intake corresponding to the average protein requirement [5,6].

Incomplete proteins are proteins that have a lower anabolic effect due to their lower digestibility, lower content of essential amino acids (especially leucine), and deficiency of other essential amino acids, such as sulfur amino acids or lysine, which means that they are not fully utilized for the synthesis of body proteins, growth needs, and maintaining nitrogen balance. Most plant-based proteins are classified as incomplete proteins due to their lower content of essential, exogenous amino acids: lysine, tryptophan, methionine, and valine. Their quantity determines the quality of a protein, consistent with the concept of a limiting amino acid, meaning one that is present in the lowest amount compared to its content in a “standard” protein [2,5,6].

In terms of achieving sustainable development goals in the food production sector, plant-based foods will become increasingly common in the market. Unlike animal protein, plant protein contains higher amounts of certain amino acids that have potentially beneficial health effects, in particular: arginine, cysteine, glutamine, and glycine. Among plant products, dry legumes, cereal products (barley, oats, corn, pasta, rice, and bulgur), walnuts, cashews, peanuts, sesame, and microalgae are characterized by a high protein content [5,6].

Numerous studies also suggest that dietary protein influences the composition and function of the gut microbiota, thereby potentially affecting the host’s health. Based on studies in vitro and conducted with animals, it was found that the process of digestion of plant proteins causes a reduction in the release of peptides and amino acids compared to animal proteins. Furthermore, it was observed that the consumption of a plant-based diet with plant-based protein correlates with an increased amount of nitrogen migrating to the colon compared to animal-based protein [7,8].

It has been demonstrated that the type of diet used has a significant influence on changes in intestinal bacterial flora. It is important to emphasize that protein is a key factor influencing the composition and function of the microbiota. Plant proteins are substrates that are available for microbial fermentation. Modification of plant protein gut bacterial metabolism leads to increased formation of short-chain fatty acids (SCFAs), which subsequently have an impact on host health. They promote the growth of the host microbiome and stimulate its activity. Additionally, it should be emphasized that many plant-based food is recognized as a source of valuable bioactive compounds classified as prebiotics (i.e., phytochemicals, vitamins, fibers, polyols, polysaccharides, and oligosaccharides). All of this together can have an impact on the long-term health of the host [9].

The aim of the review was to discuss the current state of knowledge regarding the properties of plant proteins, their impact on the intestinal microbiota, and the importance of these interactions for intestinal health in the context of the increasing role of plant-derived proteins in human nutrition.

## 2. Overview of Plant Proteins

In recent years, there has been an increasing interest in plant-based proteins as dietary components among consumers [10]. Plant proteins are a sustainable and health-promoting alternative to animal-derived proteins (weight loss and modulation of intestinal microflora). This trend is clearly reinforced by the increasing number of consumers following a plant-based diet and the noticeable shift towards plant-based protein sources [11]. Additionally, a diet rich in products with a high protein content of plant origin is recommended for people with various diseases, i.e., allergies to cow’s milk protein, or in the case of people with chronic kidney disease.

One of the most valuable criteria significant in assessing protein quality in diets is the amino acid content, the amount utilized, and the different pathways it is used for. In assessing the biological value of a protein, an approach that is used is based on comparing the content of essential amino acids in the dietary protein supply with a reference protein that meets the requirement for essential amino acids, with a protein supply corresponding to the average protein requirement. The closest to the standard protein are egg whites and breast milk [2]. In addition to high-quality protein, its digestibility is also an important evaluation parameter. Digestibility is the amount of protein consumed that can be effectively available to the body after digestion and absorption.

In 2013, FAO experts recommended a new, revised protein quality measure called digestible indispensable amino acid score (DIAAS), which is defined as DIAAS% = 100 × [(mg of digestible dietary indispensable amino acid in 1 g of the dietary protein)/(mg of the same dietary indispensable amino acid in 1 g of the reference protein)], which replaced the protein digestibility corrected amino acid score (PDCAAS). A protein DIAAS of 100% reflects that each indispensable amino acid fulfill required amounts. A DIAAS < 100% means that one or more indispensable amino acids are limited and should be supplied from other sources. It was also suggested to treat amino acids as individual nutrients because the protein digestibility does not reflect the digestibility and availability of essential amino acids. They recommend reporting, if possible, the food tables, including individual amino acid basis, which allows for assessing and classifying the quality of food protein [12].

**Figure 1:**
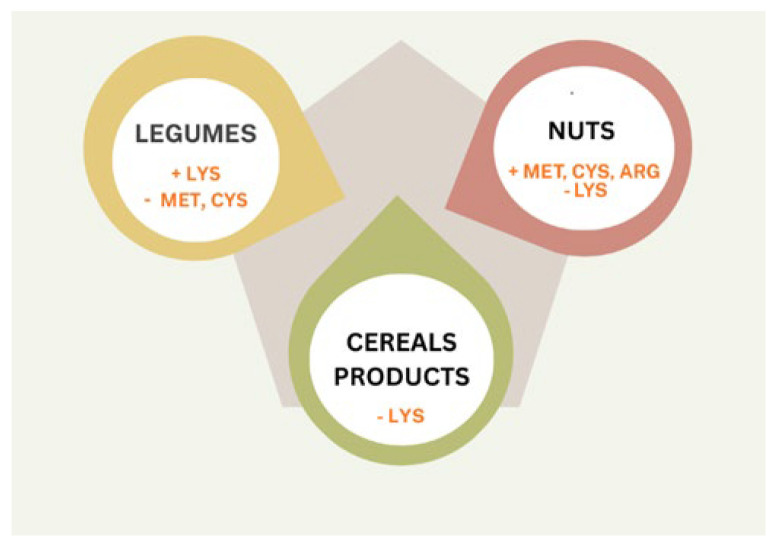
Amino acid replenishment in plant-based products.

Foods of animal origin are considered to be superior sources of protein, taking into account their complete essential amino acid composition, digestibility, and bioavailability (DIAAS above 100%). Animal protein consumption is thought to be unsustainable due to the fact that production of such foods leaves behind considerable amounts of greenhouse gas emissions, increases deforestation, and also influences water usage significantly. In this regard, protein plant production is more appropriate and sustainable. However, it has to be admitted that plant-based proteins lack essential amino acids crucial for a sustainable diet, and, furthermore, they are also less digestible (DIAS from 40% for corn and wheat to 90% for whey and soya) when compared to animal-origin products. Plant proteins generally contain a low amount of methionine, cysteine, and lysine. However, the amino acid content is different in particular plants, i.e., legumes are rich in lysine, while grains are rich in sulfur-containing amino acids [11]. This leads the researchers to believe that a nutritious plant-based diet can be obtained only if a combination of different plant-based proteins is consumed on a regular basis [13,14]. In the case of plant-derived protein, the amino acid profile and mutual proportions differ from those of animal-derived proteins. Incomplete proteins are those proteins that have a lower anabolic effect due to their lower digestibility, lower content of essential amino acids (especially leucine), and deficiency of other essential amino acids, such as sulfur amino acids or lysine, so that they are not used entirely for the synthesis of body proteins, growth needs, and maintaining nitrogen balance. Incomplete proteins include most plant-derived proteins due to their lower content of essential, exogenous amino acids: lysine, tryptophan, methionine, and valine. Their quantity determines the quality of the protein, in accordance with the concept of a limiting amino acid, i.e., one that is the smallest compared to its content in the “model” protein. Unlike animal protein, plant protein contains a higher amount of certain amino acids that potentially have health benefits, in particular: arginine, cysteine, glutamine, and glycine [15,16]. The wide variety of their sources, both conventional and new proteins, is appearing on the food market; however, it offers potential opportunities for their completeness [17,18]. Figure 1 shows an example of amino acid replenishment in plant-based products. Another strategy is the consumption of plant-based proteins with the addition of some animal proteins [6].

**Figure 2:**
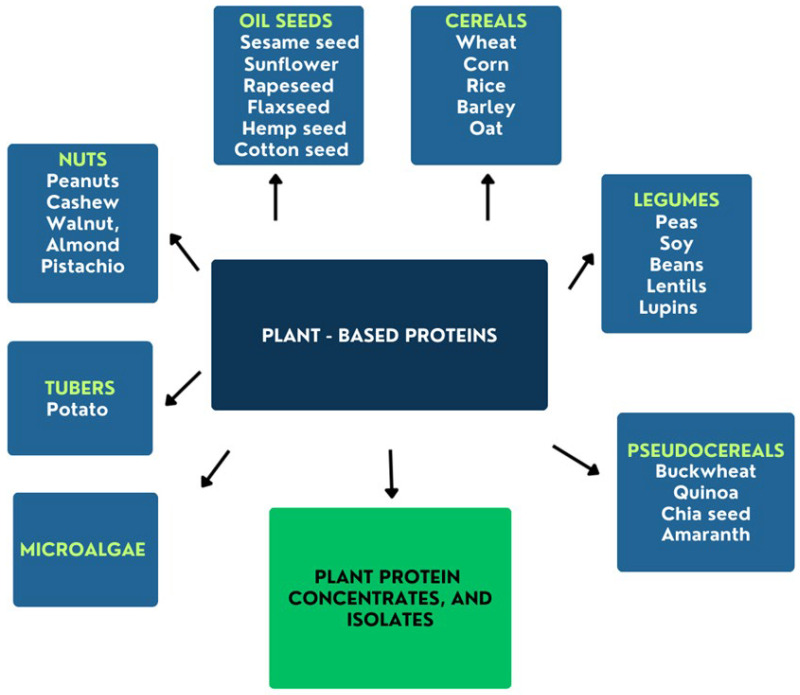
The main sources of plant–based proteins in relation to raw materials and related products.

It is estimated that 57% (wheat, maize, and rice) of worldwide dietary proteins are grains, while animal-derived proteins consist of 43% (meat, dairy, fish) [19]. Among plant products, dry legumes, cereal products, such as barley, oats, corn, pasta, rice, and bulgur, as well as proteins from walnuts, cashews, peanuts, sesame, and microalgae, are characterized by high protein content. Additionally, concentrates and isolates can also be the sources of plant-based proteins [20,21]. However, vegetables are much poorer in this component. The main sources of plant–based proteins are shown in Figure 2.

Pulses (peas, lentils, beans, and chickpeas) contain 18–32% protein, primarily albumins and globulins with different concentrations of essential amino acids. It is estimated that legumes are a quarter of worldwide crop production, with the highest levels of consumption in the Asia-Pacific region [22]. They are a source of fiber, bioactive peptides, vitamins, and minerals, along with anti-nutritional factors (trypsin and chymotrypsin inhibitors) [23,24]. Pulses contain a relatively high amount of glutamine that influences the metabolism of fat and carbohydrates [25,26]. They can lower cholesterol and triglycerides [27]. Due to the numerous benefits of pulses for food security, nutrition, health, climate change mitigation, and biodiversity conservation, there is a need to recognize their potential as part of sustainable food systems and healthy diets. The Food and Agriculture Organization (FAO) of the United Nations designated 10 February as World Pulses Day [28].

Globally, cereals (wheat, oat, rice, barley, sorghum, millet, and maize) are the main source of energy and carbohydrates, as well as the main contributor of dietary plant-derived protein, including essential amino acids. They provide more protein in the human diet than meat products [29,30]. Cereals contain from 7% to 15% of protein and provide nutrients, fiber, and bioactive compounds [28,31]. Cereal proteins mainly comprise albumins, globulins, gliadins, and glutelins. Cereal grains are rich in glutamine but have less lysine content [32]. Most cereal proteins are located in the endosperm [33]. Human health benefits of cereals have been proven when whole grains were consumed. The lower risk of developing type 2 diabetes, cardiovascular disease, colorectal cancer, and breast cancer was found along with reduced mortality [34]. Rice endosperm proteins were shown to be effective in hypercholesterolemia [35].

Worldwide, there are over 200 kinds of oilseeds and nuts (e.g., soybean, peanuts, rapeseeds, chia, flaxseed, sesame, pumpkin, sunflower). Oilseeds contain protein, fat, sugar, starch, vitamins, water, and minerals [36]. The protein content ranges from 6% to 45%, and some of them have health-enhancing bioactivity [37]. Soybeans contain 35–40% valuable protein and about 20% oil. Soybeans are a source of phospholipids, vitamins, and minerals, along with trypsin inhibitors. The soybean proteins can be an alternative to animal proteins in diet [38,39]. According to the Joint Food and Agriculture Organization of the United Nations, World Health Organization, and United Nations University (FAO/WHO/UNU) Expert Consultation on Protein and Amino Acid Requirements in Human Nutrition, the soya protein digestibility-corrected amino acid score (PDCAAS) predicts that the efficiency of protein digestibility and biological value is 0.99, which is similar to animal protein [2].

Seaweeds, especially brown, green, and red seaweeds, are mainly produced and consumed in Asia [40]. Algae contain different amounts of protein, ranging from 15% to 70% (in green *Chlorella*) of dry weight. In algae, the largest amounts of amino acids are glutamic acid and aspartic acid. Some algae species are reach source of taurine [2,25]. It is estimated that between app. 30% of algae are consumed as food, but the rest reach the food market in the form of additives or food ingredients [41].

## 3. Interactions Between Plant Proteins and Gut Microbiota

The term “microbiota” refers to the living microorganisms found in a defined environment, such as oral and gut microbiota [42]. The gut microbiota is considered the most significant factor in maintaining our health. Diet is a key factor determining which species will prevail in the microbiota [43,44]. The plant-based foodstuff is characterized by complex chemical diversity, which interacts with the metabolic activities of gut microbiota, resulting in multiple, heterogeneous responses. Zhang et al. estimate that app. 70% of gut microbial enzymes are involved in the biotransformation of plant-derived food compounds [45]. Numerous studies suggest that dietary protein influences the composition and function of the gut microbiota, thereby potentially affecting the host’s health [46]. The amount of protein in food affects how much protein reaches the large intestine, where it may undergo biotransformation carried out by microorganisms, and fermentation processes can also take place. Diets focused on plant-based products, especially those high in dietary fibers and polyphenols, are linked to an increase in beneficial gut bacteria, like *Bifidobacterium*, *Lactobacillus*, and *Faecalibacterium prausnitzii*, while also decreasing harmful bacteria [47,48].

It has been shown that a diet based on plant-based ingredients promotes a microbiota profile with a predominance of Bacteroidetes over Firmicutes bacteria, which is related to the prevention of lifestyle diseases (obesity, type 2 diabetes). This bacterial profile also reduces the formation of trimethylamine N-oxide (TMAO), a risk factor for cardiovascular diseases, due to intestinal bacteria, and this effect is further enhanced by an increased number of protective *Prevotella* bacteria, which reduce serum LDL cholesterol. A reduced number of probiotic *Bifidobacterium* bacteria was also noted, accompanied by an increased number of other bacteria with similar effects, including *Prevotella*, *Lactobacillus plantarum*, and *Streptococcus thermophilus* [49,50,51].

The vegetarian diet also demonstrated anti-inflammatory effects, which are related to the dominance of beneficial bacteria from the genera *Roseburia*, *Ruminococcus*, and *Faecalibacterium prausnitzii*, which produce SCFAs with anti-inflammatory properties. Similarly, the microbiota contained reduced amounts of pro-inflammatory bacteria from the phylum *Proteobacteria* and the family *Enterobacteriaceae* [52].

*The process of digesting proteins in the stomach and small intestine*

Gastrointestinal (GIT) digestion refers to the series of mechanical and chemical processes that break down food after it is ingested, facilitating absorption and use by the body. This entire process takes place along the digestive tract, extending from the mouth to the small and large intestines. When food is consumed, the initial breakdown occurs through chewing (mastication), which is aided by saliva and digestive enzymes. The process then progresses through various stages, including swallowing (deglutition), digestion in the stomach, secretion from the pancreas and bile, intestinal digestion and absorption in the small intestine, microbial fermentation in the colon, absorption of water and electrolytes, and finally, the regulation of the resulting food components through hormonal and neural mechanisms [53,54].

The digestion of plant proteins is a multi-step process, including reactions happening in the stomach [denaturation and primary proteolysis] and in the gut [intensive proteolysis with pancreatic enzymes and further digestion of peptides into amino acids]. In practice, plant proteins exhibit lower digestibility than those of animal origin, owing to their specific structure, connections to the cell wall matrix, and presence of anti-nutritional compounds that limit substrate availability for the digestive enzymes [55].

The primary process of protein digestion begins in the mouth. This is the place where food particles are crushed into smaller pieces and mixed with saliva, initiating the first digestive processes, and then they are swallowed and passed over for further processing. After a meal, the buffering effect of food ingredients typically causes the stomach’s pH to rise, and then gastric secretions cause it to fall. The suppression of pepsin activity by post-prandial buffering of gastric pH may be relatively transient because pepsin is most active at pH ∼ 2, but it exhibits partial activity up to pH ∼ 6. The stomach’s limited proteolysis mostly releases peptides and aromatic amino acids that notify gut sensory systems of the meal’s composition [56,57,58]. In order to bring the pH of the gastric chyme near neutral, bile, pancreatic enzymes, and bicarbonate are added in the duodenum. The ileum and jejunum get additional secretions of mucus, water, and bicarbonate [59]. Proteases in the lumen break down food protein into small peptides and amino acids as it passes through the ileum. By denaturing proteins and making them more vulnerable to proteases, physiological surfactants, like phospholipids and bile acids, also aid in proteolysis [60]. In addition to secreting vesicles containing proteases into the periapical region, the villi on the apical surface of enterocytes (the “brush border”) contain anchoring proteases [61,62].

Because some fibrous proteins are not fully digested, not all amino acids are absorbed and made available for the body to use, which reduces their nutritional value [57,63,64]. Due to their lower digestibility, lower essential amino acid content (particularly leucine), and deficiency in other essential amino acids, such as sulfur amino acids or lysine, plant proteins have less anabolic effect than animal proteins, despite the fact that plant-sourced proteins offer health and environmental benefits and are increasingly included in study formulas [65,66].

Based on in vitro studies and animal experiments, it was found that the digestion of plant proteins results in a reduction in the release of peptides and amino acids compared to animal proteins [67]. Furthermore, it was observed that consuming a plant-based diet with plant-based protein correlates with an increased amount of nitrogen migrating to the colon compared to consuming animal-based protein [68,69]. It should be noted that differences in the digestibility of plant and animal proteins arise not only from the presence of anti-nutritional compounds but also from the distinct structural properties of the proteins themselves and their organization within the food matrix. These characteristics directly determine the accessibility of digestive enzymes to peptide bonds and modulate the kinetics of proteolysis in the gastrointestinal tract [70].

At the level of secondary and tertiary structures, plant proteins are characterized by a high degree of structural order, with predominance of β-sheet conformations and strongly stabilized globular domains. This conformation promotes the formation of an extensive network of intramolecular interactions, including hydrogen bonds and hydrophobic interactions, which increase protein resistance to denaturation in the acidic environment of the stomach. As a result, potential cleavage sites for pepsin remain partially inaccessible, thereby limiting the initiation of plant protein digestion compared to animal proteins, which undergo denaturation more efficiently [71]. An additional significant factor impeding the proteolysis of plant proteins is the high prevalence of disulfide cross-links that stabilize their three-dimensional structure. Seed storage proteins, such as globulins and prolamins, exhibit extensive covalent networking, which limits their susceptibility to unfolding and reduces molecular flexibility. Consequently, pancreatic proteases, such as trypsin and chymotrypsin, encounter physical barriers to accessing peptide bonds, whereas animal proteins generally display a lower degree of covalent stabilization and a correspondingly higher accessibility of hydrolysis sites [56,72]. The next limitation specific to plant proteins is their close association with the plant cellular matrix. These proteins are often entrapped within cell wall structures rich in non-starch polysaccharides, which are not digested by endogenous human enzymes. Such physical architecture restricts the diffusion of digestive enzymes and slows the release of proteins into the intestinal lumen, resulting in delayed and incomplete proteolysis. In contrast, comparable structural barriers are largely absent in animal-derived proteins [73]. In summary, the structural aspects of plant proteins—including their stable spatial conformation, high degree of covalent cross-linking, embedding in a difficult-to-degrade cell matrix, and tendency to aggregate—significantly and specifically limit the effectiveness of digestive enzymes. Taking these mechanisms into account, in combination with the presence of anti-nutritional compounds, allows for a more detailed explanation of the mechanism of differences in the digestibility of plant and animal proteins.

*Protein metabolism by the gut microbiome*

The small intestine is where most amino acids and peptides are absorbed. However, a small amount of undigested proteins, free amino acids, and short peptides enter the large intestine, where they provide a source of nutrients for intestinal microorganisms [74]. Numerous biochemical processes, particularly fermentation, occur in the cecum and proximal colon. These processes thrive in these areas because they have a rich supply of carbohydrates and water. The cecum, which is the first part of the large intestine, and the proximal colon provide an ideal environment for various microorganisms. These microorganisms break down complex carbohydrates that are not fully digested in the small intestine, producing valuable byproducts, including short-chain fatty acids and gases. This fermentation process plays a crucial role in maintaining gut health, overall digestion, and nutrient absorption [75,76].

Fermentation in the large intestine mainly involves breaking down dietary polysaccharides, oligosaccharides, and disaccharides into simple sugars, which serve as substrates for bacterial enzymatic processes. Notably, carbohydrate fermentation in the proximal colon produces valuable short-chain fatty acids (SCFA), hydrogen (H_2_), and carbon dioxide (CO_2_). In contrast, the fermentation of amino acids or proteins results in the production of branched SCFAs, along with hydrogen, carbon dioxide, methane (CH4), phenols, and amines, highlighting the complex interactions within the digestive system [77,78,79]. Plant-derived proteins play a crucial role in shaping the gut microbiota by promoting the growth of bacteria that produce short-chain fatty acids (SCFAs), particularly butyrate and acetate. This mechanism arises from the combination of the chemical properties of the protein fractions and their co-occurrence with fiber and other bioactive components [80].

Acetate represents a significant short-chain fatty acid (SCFA) predominantly found in the large intestine, constituting over fifty percent of all SCFAs identified in fecal matter. The production of acetate by gut microbiota occurs primarily through two metabolic pathways, one of which is the fermentation of carbohydrates by intestinal bacteria. The second one is via the Wood–Ljungdahl pathway, where approximately one-third of acetate originates from acetic acid bacteria, which are capable of synthesizing it from hydrogen and carbon dioxide, or from formic acid [81,82,83,84]. Butyrate is synthesized via two pathways: a kinase pathway, involving phosphotransbutyrylase and butyrate kinase to convert butyryl-CoA into butyrate, and/or as a result of the transformation of butyryl-CoA into butyrate through a single enzymatic reaction carried out by bacteria producing butyrate [85]. In the gut microbiome, a process known as “cross-feeding” commonly takes place, where the byproducts of one group of bacteria are utilized by another group to produce different metabolites. This type of cross-feeding with short-chain fatty acids (SCFAs) typically occurs from acetate to butyrate, and to a lesser extent, involves propionate as well [86,87].

Several studies [74,88,89,90] have thoroughly examined and underscored the critical role that short-chain fatty acids (SCFAs) play in promoting gastrointestinal (GI) and metabolic health, revealing intricate mechanisms that contribute to these health outcomes. SCFAs, which include acetate, propionate, and butyrate, are produced through the fermentation of dietary fibers by gut microbiota and exhibit a broad spectrum of beneficial effects that are essential for maintaining overall human well-being. Their actions are primarily mediated through two distinct yet interconnected pathways.

The first pathway highlights the interactions of SCFAs with G protein-coupled receptors (GPCRs), a vast family of receptors located in various organs, including the intestine, kidneys, heart, and adipose tissue. These GPCRs are expressed in numerous cell types throughout the gastrointestinal tract, including enterocytes, enteroendocrine cells, immune cells, and neuronal cells, and they play a crucial role in mediating physiological responses. When SCFAs bind to these receptors, they activate signaling cascades that can influence processes, such as inflammation, insulin sensitivity, and gut motility, thereby promoting overall digestive and metabolic health [91,92,93].

The second pathway focuses on the role of SCFAs as inhibitors of histone deacetylases (HDACs). By inhibiting these enzymes, SCFAs promote the acetylation of histones, leading to increased gene expression associated with key cellular functions. This regulatory mechanism affects several biological processes, including cell metabolism, differentiation, and proliferation. Enhanced expression of specific genes can lead to improved gut barrier function and immune responses, contributing to a healthier intestinal environment [94,95,96].

A deeper investigation into the diverse roles of short-chain fatty acids (SCFAs) is necessary to fully understand their potential impact on health maintenance and disease prevention. As research progresses and unveils the complexities of SCFAs along with their beneficial effects, it becomes evident that they offer significant promise for enhancing gastrointestinal (GI) and metabolic health.

## 4. Health Implications

It is well known that food consists of nutrients and other constituents that can modify the host gut microbiota. Imbalances in the intestinal microflora, also known as dysbiosis, are often associated with the development of various diseases, including irritable bowel syndrome, functional gastrointestinal disorders, atopic dermatitis, and depression. It is also suspected that dysbiosis may increase the risk of certain lifestyle diseases, including type 2 diabetes, obesity, and hypertension. Currently, new factors responsible for changes in the composition and functioning of the intestinal microflora are being sought. It has been demonstrated that the type of diet used has a significant influence on changes in intestinal bacterial flora [97,98,99,100]. There is accumulating evidence that suggests protein is an essential driver of microbiota composition and function and that microbiota–protein interactions may have significant impacts on long-term host health [42]. On the other hand, Staudacher and Loughman [101] present the term “gut health” as the absence of gastrointestinal symptoms and disease, as well as the absence of other unfavorable local conditions, including increased intestinal permeability, mucosal inflammation, or deficiency of short-chain fatty acids (SCFAs).

It is well known that the digestibility of plant-derived protein is limited. Additionally, the nonprotein components of plant foodstuffs can diminish digestibility. Generally, the availability of plant protein absorption depends on factors, both external (undamaged cell structures with entrapped proteins, anti-nutritional compounds, such as protease inhibitors, i.e., phytates and trypsin inhibitors, and fibers) and internal (profiles of amino acid, protein folding, and cross-linking) [102]. When not digested, proteins, peptides, and amino acids, along with carbohydrates and fat, reach the colon and become substrates for microbial metabolism. Plant proteins are substrates that are available for microbial fermentation. They are an important source of carbon and nitrogen for the living microorganisms of the large intestine. Due to the limited digestibility of plant proteins and, therefore, their higher availability for colon microbial bacteria metabolic processes, the gut nitrogen content in the large intestine is higher. In this regard, it is important to emphasize that nitrogen, as a limiting nutrient, may have an impact on colon microbial composition and metabolism. Thus, undigested dietary protein provides additional nitrogen, which can stimulate growth of some species, i.e., *Bacteroidales* and *Clostridium* [103]. Modified by plant protein, gut bacterial growth and metabolism (*Bacteroidales*) lead to increased formation of fermentation byproducts (i.e., short-chain fatty acids and nitric oxide), which subsequently have an impact on host health [46,103]. Many studies confirmed that dietary plant proteins promote the diversity of the gut microbiome [104]. Plant proteins promote the growth of probiotic genera *Lactobacillus* and *Bifidobacterium*, as well as *Akkermansia*, which is classified as the next generation of probiotics. The gut abundance of this bacterial genus has a favorable impact on metabolic syndrome, reduces overweight and obesity, improves cholesterol metabolism, reduces oxidative stress, strengthens the intestinal barrier, and modulates inflammation [104]. However, it should be emphasized that the plant protein modulation of the composition of the colon microbiota depends on the plant genus. In the study of Mancabelli et al., it was found that yellow pea (*Pisum sativum*) extracted protein increases gut abundance of *Bifidobacterium longum* and *Faecalibacterium duncaniae* and decreases *Bacteroides*, *Parabacteroides*, and *Phocaeicola* [105]. The effects of different plant proteins on the microbiota were evaluated by Zhao et al. [9]. The model INFOGEST, which simulates the digestive process in the human upper gastrointestinal tract, was used to test the influence of six plant protein isolates (pea, chickpea, mung bean, soybean, lentil, and brown rice) and a milk protein on the microbial composition. The collected protein residues were co-fermented with wheat arabinoxylan. Brown rice, chickpea, and pea protein were shown to increase the abundance of *Bacteroides plebeius* (family of *Bacteroidetes)* by 45–50%. Treatments with pea, chickpea, lentil, and milk protein were shown to decrease the abundance of the *Lachnospiraceae* family (*Blautia, Eubacterium*, and *Fusicatenibacter*) and to increase *the Lachnoclostridium.* Within the *Firmicutes* genus, the response was diversified. The treatment with mung bean and brown rice protein increased the abundance of *Megasphaera* and *Megamonas genera*. Chickpea and lentil protein treatments increased the genus of *Phascolarctobacterium*. Additionally, the authors found that all tested proteins increased propionate synthesis from 17% to 25–32% [106].

In the American Gut Project (11,336 participants), McDonald et al [107], compared consumers eating more than 30 types of plants with those who consume 10 or fewer plants per week. It was shown that short-chain fatty acid fermenters, species *Faecalibacterium prausnitzii* and genus *Oscillospira*, were increased in stool samples of individuals who consumed more plants [107]. Plant proteins promote the growth of the host microbiome, stimulate its activity, and directly confer health benefits to the consumer [105]. Additionally, it should be emphasized that many plant-based foods are recognized as a source of valuable bioactive compounds classified as prebiotics (i.e., phytochemicals, vitamins, fibers, polyols, polysaccharides, and oligosaccharides) [107,108]. So, the host health benefits of plant proteins are mainly due to active peptides and the modification of intestinal microbiota. Cereal and legume proteins, by modification of gene expression, may favorably influence cholesterol metabolism (hypocholesterolemic effect) and glucose homeostasis [109,110]. In the study by Li et al., pea protein was shown to reduce systolic blood pressure, which was attributed to the reduction in angiotensin II level by bioactive pea peptides [111]. Plant proteins can exhibit anti-inflammatory effects and alleviate symptoms of intestinal diseases by modulating gut microbial metabolites [111,112,113].

According to David et al., the human gut microbiota is flexible and can be modulated according to the type of diet: herbivorous or carnivorous. This quick reshape of the human microbiome reflects the evolutionary adaptation to seasonal changes in available nutrition [114]. In the study by Budhathoki et al. [115]. 70,696 Japanese adults were followed up for a mean of 18 years to assess the differences between animal and plant protein intake on all-cause and cause-specific mortality. The study showed that a high-protein plant diet was associated with decreased total mortality and cardiovascular-related mortality. Additionally, it was found that the substitution of animal protein by plant protein lowered the risk of total cancer-related mortality. The authors concluded that 3% energy equivalent of red meat protein replaced by plant protein would reduce overall mortality by 3.60% after 15 years. Another study of the Nurses’ Health Study and the Health Professionals (131 342 participants) conducted by Song et al. [116], confirmed that a plant protein diet is associated with lower all-cause mortality and cardiovascular mortality. It was estimated that the replacement of an equivalent amount of 3% energy by plant-derived protein would result in a 10% decrease in overall mortality and a 12% decrease in lower mortality due to cardiovascular mortality. The positive impact of plant protein intakes was reported on blood pressure, lipid profile, irritable bowel syndrome, obesity, and diabetes mellitus [117,118]. Health benefits of a plant protein diet were also confirmed in a meta-analysis. Zheng et al. [119] analyzed 12 studies with a total of 1,450,178 subjects (mean follow-up of 7 years) and found an inverse association of isocaloric meat substitution by plant protein on all-cause mortality and cardiovascular mortality Similar results were reported by Naghshi et al. [120] from the meta-analysis of 31 studies of a total of 715,128 participants (follow-up from 3.5 to 32 years). Plant protein intake was associated with a reduced risk of all-cause mortality and cardiovascular diseases. Although the authors found that a high intake of total proteins was associated with a lower risk of all-cause mortality, this intake of animal protein was not associated with reduced mortality from cardiovascular disease or cancer. Therefore, it may be recommended to increase plant protein consumption in the general population.

Many beneficial health effects of phytonutrients mediated by the gut microbiome were confirmed; however, the specific clinical outcome of plant proteins varies between the types of plants used. In the human diet, the main substantial source of plant-derived proteins is grains and legumes (i.e., soy, pulses, pea, chickpea, lentil), while oil crops, nuts, seeds, vegetables, and aquatic plants, like algae, are less important but promising sources [121]. Soya proteins were found to have a positive impact on the gut by lowering the Firmicutes/Bacteroidetes ratio (F/B), which is recognized as an indicator of gut health associated with obesity [122]. Metaproteomics analysis showed that Bacteroidetes growth is supported by higher nitrogen availability, which can be supplied with a plant protein diet [123,124]. Plant-derived proteins also promote the growth of beneficial microorganisms, like *Akkermansia* and *Bifidobacterium*, and also enhance the production of SCFAs by the gut microbiome [112,125]. In the study by Govindan et al., the diet of healthy volunteers supplemented with lentil protein enhanced microbiome diversity and promoted growth of SCFA-producing bacteria with an increase in SCFAs [126]. According to the results of the study by Beaumont et al., soy protein cannot only modify gut microbiota but also change the expression of host genes responsible for rectal mucosa homeostasis. They also compared animal and plant protein influence on the gut bacterial metabolic profile and found the dependence of protein source [100]. SCFAs, through intracellular signaling and gene expression modulation, have properties of lowering lipids and preventing obesity, and they can influence immune cell proliferation [104].

Regarding the complexity of plant protein biotransformation by the gut microbiome, it should be noted that some metabolites may affect host health negatively. Whereas deamination of all amino acids increases luminal concentrations of H_2_, CO_2_, and ammonia, microbial decarboxylation of amino acids produces biogenic amines that are basic compounds with high polarity. Gut microbes produced biogenic amines (histamine, cadaverine, putrescine, serotonin, tryptamine, tyramine, spermine, and spermidine). The role of biogenic amines is still unclear, but they may induce diarrhea or be mediating factors in host–microbe interaction, contributing to immunomodulation [127,128]. 

Conditions favorable to the production of biogenic amines are associated with natural microbiological and biochemical processes in food. A balanced perspective is not about completely eliminating these processes but about consciously controlling them. Integration of issues across food technology, microbiology, and environmental aspects enables reducing health risks while maintaining the safety and high-quality principles of sustainable food production. Biogenic amines are low-molecular-weight nitrogen compounds formed mainly by microbial decarboxylation of amino acids, which is influenced by amination and transamination of aldehydes and ketones [129]. 

The basic condition for the formation of biogenic amines is the presence of free amino acids such as histidine, tyrosine, ornithine, or lysine. Their increased availability results from intense proteolytic processes during maturation, fermentation, or uncontrolled spoilage of raw materials. While these processes are desirable for shaping the sensory characteristics of food, they can also lead to excessive accumulation of biogenic amines if not properly controlled [130]. 

It should be noted that microorganisms with amino acid decarboxylase activity play a key role in the production of biogenic amines, including bacteria of the genera *Enterococcus*, *Lactobacillus*, *Pediococcus*, *Morganella*, and *Enterobacter*. Conditions favorable to their development, such as insufficient raw material hygiene, secondary contamination, or lack of control of the fermentation microflora, significantly increase the risk of amine accumulation. A sustainable technological approach involves selecting starter cultures that do not synthesize biogenic amines and adopting good production practices to reduce their formation, without eliminating traditional food production methods [131,132].

Environmental parameters, such as temperature, pH, water activity, salt concentration, and oxygen availability, can also significantly affect the intensity of biogenic amine production. The rate of accumulation is significantly affected by higher storage and processing temperatures, a favorable pH for decarboxylase activity, and long fermentation or storage times. Optimizing these parameters to ensure product safety and reduce energy and raw material losses is crucial for sustainability, e.g., through rational cold chain management [132,133].

The dietary pattern significantly determines both the types of proteins consumed and the technological and environmental factors involved in their production, which favor the formation of biogenic amines. In this case, the Mediterranean diet and the Western model of nutrition (USA) exhibit significant differences. From the perspective of biogenic amine production, the Mediterranean diet provides proteins with a moderate content of free amino acids. Promoting more diversity in the gut microbiota restricts the dominance of microorganisms with high decarboxylase activity [134,135]. The growth of bacteria that produce biogenic amines is limited and inhibited by the presence of bioactive compounds (such as polyphenols) and fiber. As a result, the conditions conducive to excessive biogenic amine production are relatively limited in this nutritional model, consistent with the concept of a sustainable and safe food system based on plant proteins [136]. 

Many cited study results confirmed the reduced mortality of dietary plant protein, and, therefore, its beneficial role on human health should not be questioned. Additionally, plant protein consumption reduces the occurrence and growth of colon, cervical, and breast cancer. It has anti-inflammatory, antioxidant, and antiproliferative potential and has a positive impact on hypertension, metabolic disorders (hyperlipidemia, obesity, diabetes mellitus), and irritable bowel syndrome [137,138]. Yet, it is unknown how biogenic amines function physiologically in the large intestine. On the one hand, polyamines are crucial for intestinal growth and differentiation as well as the immunomodulation of the human microbiota [127]. Given that aromatic monoamines (dopamine, serotonin, tryptamine, tyramine, and 2-phenylethylamine) function as neurotransmitters and may have a signaling role in the colon, the microbial catalytic activity of some L-amino acid decarboxylases may be advantageous. It is hypothesized that a microbe-derived tryptamine may increase intestinal secretion [139] However, endogenous nitration of amines results in nitrosamines, which are linked to altered DNA structure in colonocytes and enhanced fecal water genotoxicity, raising the risk of colorectal cancer (CRC) [140].

There is much evidence that plant protein modifies gut microbiome and gut bacterial metabolic profile and has an impact on homeostasis; subsequently, health data from human studies are limited. To explain the essential interaction between plant protein and microbiome and assess health impact, further scientific research is needed, both in healthy and ill patient populations.

**Figure 3:**
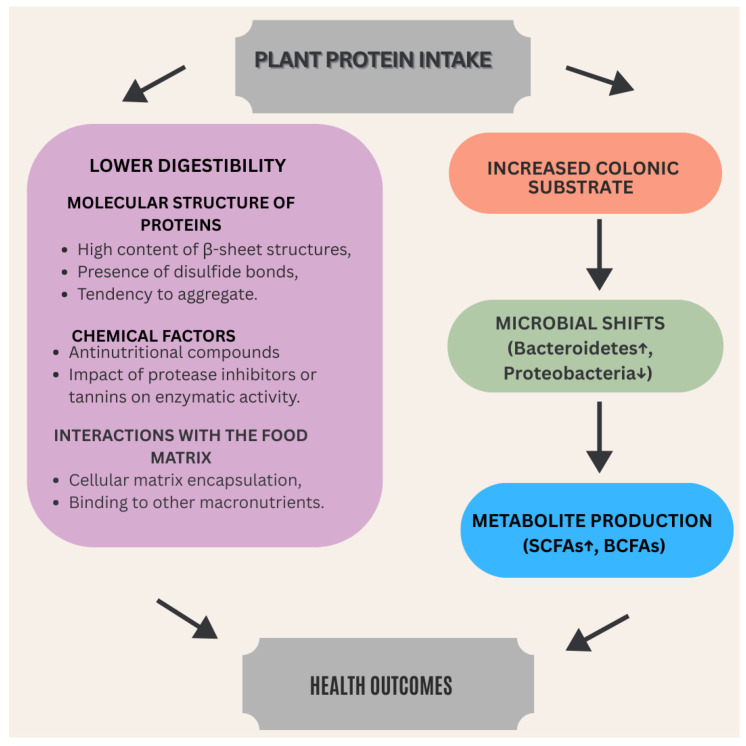
The scheme of summarizing interactions between plant proteins and gut microbiota.

A schematic summarizing the main narrative of the presented review is shown in Figure 3.

Interactions between plant-derived proteins and the gut microbiota constitute a multifaceted and tightly regulated component of host–diet–microbe crosstalk. Accumulating evidence indicates that the structural features and digestive behavior of plant proteins influence the extent and form of nitrogen delivery to the colon, thereby shaping microbial composition and metabolic functionality. These processes affect the production of microbial metabolites with potential physiological relevance for the host.

## 5. Challenges and Future Directions

To assess the role and significance of plant protein in human nutrition and to understand the influence on gut microbiota, in further research, the identification and quantification of multiple diet proteins with metaproteomics techniques seems to be an effective and advantageous strategy. These analyses may provide information about the interaction of plant proteins within a microbiome. The information on host microbiome composition, abundance, metabolism, and gene expression responses according to varying conditions can also be obtained [141]. The techniques of labeling with heavy carbon or nitrogen, i.e., the direct protein stable isotope fingerprint (SIF) method, allow for the tracing of metabolic substrate [142,143]. SIF allows the assignment of specific proteins to specific microorganisms, therefore identifying the dietary protein as substrates consumed by gut microorganisms [144].To clarify many issues related to the dietary plant-derived proteins’ impact on gut microbiota (i.e., microbiome homeostasis, metabolite production, host gene expression), including their consequences for human health, there is a great need for more studies in various populations, from infants to the elderly and healthy and ill patients. Additionally, the studies that isolate the effect of plant protein from other dietary plant-derived components (fibers, polyphenols, vitamins, minerals) to establish direct causality should be scheduled. There is still a lack of data showing the association between human health benefits of plant protein intake in comparison to animal protein. It is well known that animal and plant foods vary in protein and amino acid content. According to digestibility and amino acid composition (including content of essential amino acids), animal proteins have a higher quality than plant proteins [6,145]. Therefore, it is crucial and recommended that plant protein in the diet should be diverse to supply. To assess effects or establish dietary recommendations, large and long-term studies are required. The study by Huang et al., 2020 [145] of the US population included 416,104 men and women, with a 16-year follow-up. It was found that greater intake of plant protein from various sources was significantly associated with lower overall mortality and cardiovascular disease mortality. The results were independent of other factors (smoking status, diabetes, fruit consumption, vitamin supplement use, and self-reported health status). The replacement of 3% of energy animal-derived protein with plant protein reduced overall mortality by 10% and cardiovascular disease mortality by 11% in men and 12% in women. The authors concluded that “Findings from this and previous studies provide evidence that dietary modifications in choice of protein sources may promote health and longevity” [146]. However, the discussion of the underlying mechanisms contributing to lower mortality of plant proteins due to heterogeneity of other nutrients is still open [147,148]. The potential mechanisms include the difference of amino acid content between animal and plant proteins (higher content of nonessential amino acids arginine and glycine in plant-derived proteins with fewer indispensable amino acids: methionine, lysine, and tryptophan) and high content of bioactive compounds in phytonutrients [149]. To confirm that diets enriched with plant protein have an impact on colonic microbiome and on gut epithelium homeostasis and functions resulting in health benefits and longevity, the underlying mechanisms should be elucidated and tested in further experimental studies.

## 6. Conclusions

The analysis of the literature on the subject suggests that plant-derived proteins play a significant role in shaping the composition and metabolic activity of the intestinal microbiota. The digestive process in the stomach and intestines reveals the impact of different types of diets (plant or animal origin) on the gut microbiota. The nature of this interaction is influenced by factors such as protein structure, amino acid profile, and interactions with accompanying fractions present in the plant matrix, i.e., dietary fiber, polyphenols, and the composition or type of product processing. An important issue is the lower digestibility of plant-derived proteins compared to animal-derived proteins, which results in increased availability of protein substrates for the gut microbiota. Interactions between plant proteins and the gut microbiota influence the production of microbial metabolites, including short-chain fatty acids, branched-chain fatty acids, and nitrogen compounds, which can exert both beneficial and potentially adverse physiological effects in the host. The research results suggest that diets rich in plant-based proteins promote an increased proportion of saccharolytic and potentially health-promoting bacteria while limiting the growth of proteolytic bacteria associated with inflammatory processes. In terms of achieving sustainable development goals in the food production sector, plant-based foods will become increasingly common in the market. This poses a challenge for interdisciplinary teams of food technologists, microbiologists, and dietitians in their pursuit of developing innovative functional products with the addition of plant-based proteins. Promoting information about the actual beneficial effects of such a product on health and consumer acceptance also remains an important issue. Despite the growing interest in plant-based diets, there is still no information in the literature on the impact of high plant protein intake on the gut microbiota and human health. Understanding the nature of such mechanisms, taking into account the level of plant protein intake, could be used in the development of personalized nutritional strategies.

## Data Availability

Data are contained within the article.
